# Prolonged Intermittent Trunk Flexion Increases Trunk Muscles Reflex Gains and Trunk Stiffness

**DOI:** 10.1371/journal.pone.0162703

**Published:** 2016-10-21

**Authors:** Matej Voglar, Jeffrey Wamerdam, Idsart Kingma, Nejc Sarabon, Jaap H. van Dieën

**Affiliations:** 1 University of Primorska, Andrej Marušič Institute, Koper, Slovenia; 2 MOVE Research Institute Amsterdam, Department of Human Movement Sciences, Vrije Universiteit Amsterdam, Amsterdam, The Netherlands; 3 S2P Ltd., Laboratory for Motor Control and Motor Learning, Ljubljana, Slovenia; Northwestern University Feinberg School of Medicine, UNITED STATES

## Abstract

The goal of the present study was to determine the effects of prolonged, intermittent flexion on trunk neuromuscular control. Furthermore, the potential beneficial effects of passive upper body support during flexion were investigated. Twenty one healthy young volunteers participated during two separate visits in which they performed 1 hour of intermittent 60 seconds flexion and 30 seconds rest cycles. Flexion was set at 80% lumbar flexion and was performed with or without upper body support. Before and after intermittent flexion exposure, lumbar range of motion was measured using inertial measurement units and trunk stability was assessed during perturbations applied in the forward direction with a force controlled actuator. Closed-loop system identification was used to determine the trunk translational admittance and reflexes as frequency response functions. The admittance describes the actuator displacement as a function of contact force and to assess reflexes muscle activation was related to actuator displacement. Trunk admittance gain decreased after unsupported flexion, while reflex gain and lumbar range of motion increased after both conditions. Significant interaction effects confirmed a larger increase in lumbar range of motion and reflex gains at most frequencies analysed following unsupported flexion in comparison to supported flexion, probably compensating for decreased passive tissue stiffness. In contrast with some previous studies we found that prolonged intermittent flexion decreased trunk admittance, which implies an increase of the lumped intrinsic and reflexive stiffness. This would compensate for decreased stiffness at the cost of an increase in cumulative low back load. Taking into account the differences between conditions it would be preferable to offer upper body support during activities that require prolonged trunk flexion.

## Introduction

Low-back pain (LBP) is the most prevalent musculoskeletal pain. It affects both genders across all ages with peak prevalence between 45 and 59 years of age [1]. Furthermore, LBP is the leading musculoskeletal cause for consulting a family physician and is responsible for the highest amount of years lived with disability [2]. As a result, LBP presents a considerable social and economical burden [3,4]. Several biomechanical risk factors for work-related LBP have been identified, among which frequent or prolonged bending is consistently recognized as harmful [5,6]. Nevertheless, a recent review article concluded that there is insufficient evidence for causality of this traditionally accepted occupational risk factor [7]. This conclusion, which has been heavily debated [8,9], was made in view of lack of evidence on biological plausibility in the literature included in this review. Biological plausibility here refers to the mechanism by which a risk factor might contribute to the development of back pain.

There is a growing body of evidence from animal and human studies indicating unfavourable effects of repeated and sustained flexion of the trunk on passive structures and on motor control [10–13]. It has been shown that trunk flexion induces creep deformation of viscoelastic structures, which results in reduced intrinsic stiffness of the spine [14,15]. Two kinds of spinal passive tissue loading during sustained trunk flexion have been differentiated: (i) creep loading, where an increase in deformation of the passive viscoelastic structures occurs under a constant load, and (ii) stress-relaxation, where a decrease in stress experienced by the viscoelastic materials occurs under a constant deformation. As a result of stress relaxation, when a constant flexed posture is required, stress in the passive tissues gradually decreases, requiring a shift in force distribution from passive to active structures. Both creep and relaxation involve time-dependent changes in the mechanical properties of the passive viscoelastic tissues [14,16,17]. In line with this, Olson, Li and Solomonow [18] showed that passive trunk flexion induces sustained deformation of the passive tissues. The magnitude of these tissue changes depends on factors such as external load [17], flexion rate [19], flexion angle [14], sex and age [20]. The majority of the studies investigating the effects of trunk flexion have used creep deformation loading. Such loading is indeed present during several occupational activities that require full spinal flexion. However, in occupational settings, less than full flexion is often required, which can also have effect on tissue mechanical properties if the posture is maintained. For example, Sánchez-Zuriaga and colleagues [11] showed that one hour of sustained supported sitting with 70% of lumbar flexion induced viscoelastic deformation of passive tissues. Hendershot and colleagues [14] further showed that the decrease in intrinsic trunk stiffness increased with increasing flexion angle.

In parallel with mechanical changes, several alterations in neuromuscular control were shown during and following cyclic or continuous trunk flexion. Several animal studies showed that applying a constant load directly to the ligaments decreases reflexive activation of surrounding muscles [13,21]. In line with this reduction in excitability, Sánchez-Zuriaga and colleagues [11] showed in humans that one hour of sustained supported sitting with 70% of lumbar flexion induced a significant increase of reflex onset delays. However, perturbation studies in humans indicated increased reflex gains following trunk flexion [14,22] and increased trunk extensor activity after repetitive passive flexion [18], possibly compensating for reduced intrinsic stiffness.

Simultaneous investigations of mechanical and neuromuscular control changes following trunk flexion are scarce. Furthermore, there is a lack of studies investigating the effects of longer lasting flexion exposure. Therefore, the aim of this study was to assess the effect of 60-minutes intermittent trunk flexion exposure, imitating the work of a crane operator [23]. This specific occupational group has been shown to be at increased risk for low back problems [24] potentially due to high cumulative low back loading [25]. Additionally, the goal was to assess the potential beneficial effects of a passive support of the upper body in the flexed posture. The conditions resemble stress relaxation, where the unsupported condition is more demanding for the trunk muscles, while both conditions cause similar loading of the ligaments. We hypothesised that intermittent flexion results in a viscoelastic deformation of spinal tissues, reflected in an increase in flexion range of motion and more so without passive upper body support. Furthermore, we hypothesized that reflex gains would be increased after repetitive trunk flexion, to compensate for reduced stiffness due to viscoelastic deformation, but that trunk posture would be controlled less effectively resulting in an increased admittance gain, specifically after unsupported flexion in view of muscle fatigue.

## Materials and Methods

### Participants

Participants were recruited as a convenience sample by means of personal communication and social media. Twenty-five subjects responded to the invitation from which two subjects did not complete the measurements due to technical reasons and one subject was rejected based on the exclusion criteria. One participant was later excluded due to inability to assume the required posture. Consequently, twenty-one young volunteers were included in the present study (11 males, 23.2 (2.0) years, height 182.3 (6.2) cm and body mass 73.9 (8.2) kg) and 10 females, age 24.3 (4.0) years, height 168.3 (7.2) cm and body mass 62.1 (9.0) kg). Exclusion criteria were either LBP within the last six months or any history of LBP that required at least one day of adjusted daily activities. Participants with any known sensory or neuromuscular pathologies that could affect postural control were also excluded. The research was approved by the Ethics committee for Movement Sciences (Ethische Commissie Bewegingswetenschappen) at the Vrije Universiteit, Amsterdam (approval number: ECB 2015–18). All subjects signed an informed consent statement prior to the experiment. The study was conducted in line with Helsinki Declaration recommendations.

### Experimental procedure

**Fig 1:**
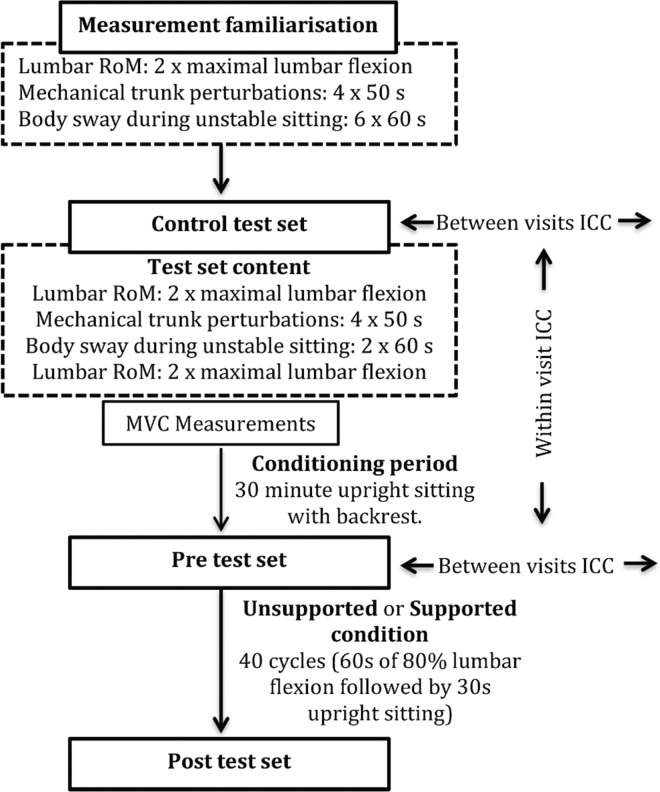
Flow chart presentation of the measurements. Each of the two visits contained the same testing protocol with the sole difference being the flexion (i.e. intervention) condition. Measurements of body sway during sitting on unstable surface were done but are not included in the paper.

Participants were invited for two visits with two different exposure conditions: supported flexion (SF) and unsupported flexion (USF). Each visit consisted of an introductory test set and three repeated identical sets of tests: control, pre-exposure and post-exposure test set (Fig 1). The control test set and a subsequent conditioning period were introduced given the results of a pilot study in which indicated a potential effect of repeated measurements on the range of motion (RoM). The conditioning period required subjects to sit for 30 minutes in a standardised position on an office chair with their backs supported against the backrest, to reduce the potential effects of earlier activities. The control test set and the pre-exposure test set were used to assess reliability of the measurements within and between visits.

Each set of tests included measurements of maximal lumbar RoM, measurements of trunk neuromuscular control during mechanical perturbations, measurements of muscle activation in response to a constant load during 2 seconds prior to the mechanical perturbations, and measurements of postural control while sitting on an unstable surface. Participants performed a standardised warm-up (20 times alternating high knee lifts, 10 times mid-range forward and backwards pelvis tilts in seated position, 3 times 3 s forward planking, and both sides lateral planking with extended arms on a 40 cm raised surface). Next, a 30-minute conditioning period was imposed, during which subjects were sitting upright with their back supported. Subsequently, after the pre-exposure test set, one of the experimental conditions was applied, consisting of 1 hour of supported or unsupported intermittent flexed sitting. Experimental conditions were introduced on separate visits in counterbalanced order with at least 4 days between visits to reduce potential carryover effects. After the experimental condition, tests were repeated (post-exposure test).

### Repetitive trunk flexion

**Fig 2:**
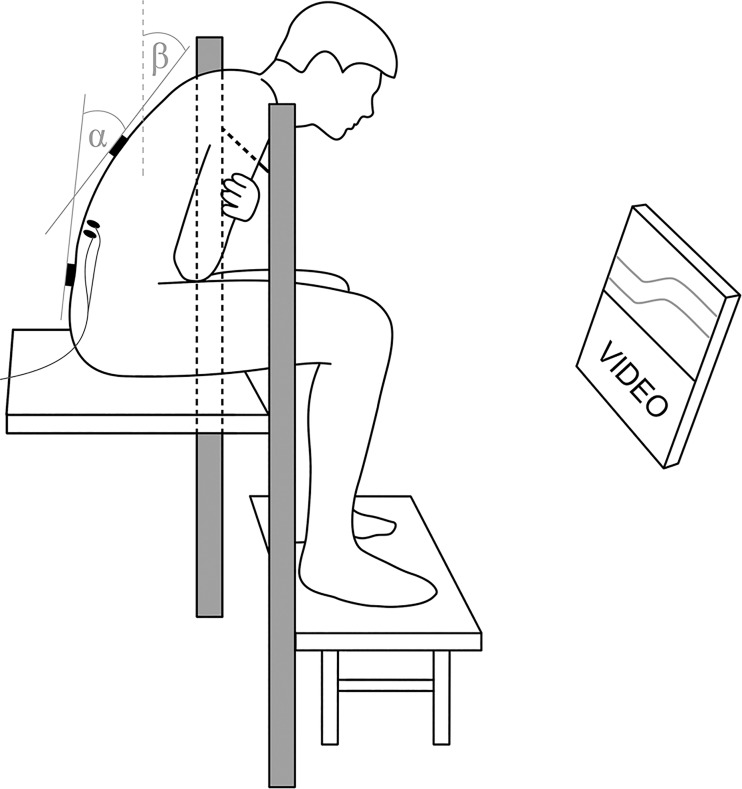
Position of the participant during intermittent flexion. Visual feedback was provided to the participant about the lumbar flexion (α) and trunk inclination (β) with marks for the required position. In the unsupported condition, a thin rope was placed horizontally in front of the participant at the appropriate height to serve as the orientation point during the flexion and in the supported position the rope was replaced by a padded bar on which the participant was leaning during the flexion.

In both experimental conditions, the participant was seated on a raised platform with the feet supported and real time feedback on lumbar flexion (the inclination difference between the sensors) and trunk inclination (inclination of the sensor over T12) was provided. The target lumbar flexion angle was determined as 80% from erect stance to maximal forward flexion, similar as in the study by Sánchez-Zuriaga and colleagues [11]. To determine erect posture, participants stood by the doorframe touching it with the right heel, the right gluteus maximus and the right scapula, leaving the sensors untouched. The maximal flexion RoM was measured as described in the next paragraph. Intermittent flexion (40 cycles including 1 minute of target flexion and 30 s of upright active sitting, cumulatively lasting for 60 minutes) was imposed. During this time videos were shown to the participants, while an audio signal indicated the time to change position. To standardise loading and avoid that subject would obtain lumbar flexion by slumped sitting, we controlled both trunk inclination and lumbar flexion. To determine the target posture, participants flexed forward until trunk inclination reached 35° and then adjusted lumbar flexion by tilting the pelvis forward or backwards to reach 80% of lumbar flexion RoM, while maintaining 35° of inclination of the sensor at T12. If in this position a participant presented with electromyography (EMG) silence of back muscles due to the flexion relaxation phenomenon, only the lumbar flexion angle was reduced until marked activation could be seen in both conditions. In the unsupported flexion (USF) condition, a thin rope was placed horizontally to provide the participant with a mechanical orientation to indicate the required trunk inclination of 35°. Participants had their hands crossed across the chest and they were touching the rope slightly with their shoulders. In the supported flexion (SF) condition the trunk inclination and lumbar flexion were obtained as described above, but the rope was replaced with a padded bar which provided passive support (Fig 2). The participants leaned on it with their chests and shoulders, while their hands were crossed on the front side of the bar. Participants were reminded to adjust position if they drifted more than 2° from the goal position as checked by feedback on the computer screen. In both conditions the activation of erector spinae pars longissimus (ESL) and pars iliocostalis (ESIC) was measured during the 1^st^ and every 4^th^ subsequent flexion cycle by means of surface EMG, resulting in 11 measurements.

### Muscle activity

Muscle activity was assessed using surface EMG (REFA, TMSi, Netherlands). Following skin preparation (shaving and cleaning with alcohol), single use, self-adhesive electrodes (Blue Sensor N, Ambu A/S, Ballerup, Denmark) were placed bilaterally over the erector spinae muscle pars lumborum (ESL; 3 cm lateral to interspinous space between L4 and L3) and pars iliocostalis (ESIC; 6 cm lateral to the L2 spinous process) [26,27]. The EMG signals were sampled at 2000 samples/s, band-pass filtered 5 – 400 Hz (2^nd^ order Butterworth), rectified and normalised to the maximal voluntary contraction (MVC) level. MVC was assessed in prone position with the upper body over the edge of the table. The participant was fixed over the ankles and distal part of the thigh and instructed to resist a downward manually applied force as strongly as possible. The experimenter gradually applied manual force bilaterally over the superior part of the scapula in anterior direction and held it for 3 s. This was repeated 3 times and a sliding window (1 s wide) was used to determine the highest muscle activity. To calculate the mean amplitude, the rectified EMG signal was further filtered using a 2^nd^ order low pass (2.5 HZ) Butterworth filter to obtain a linear envelope, followed by MVC normalisation and averaging over the time frame. For the calculation of the median frequency, the raw EMG was band-stop filtered at 50 Hz to reduce the hum artefact and the power spectrum was calculated using fast Fourier transformation. For both mean amplitude and median frequency parameters two seconds time windows during constant force application were used and the results were averaged over four repetitions within each test set.

### Lumbar flexion measurements

Pelvis and thorax orientations were estimated using two inertial measurement units (IMU) with six degrees of freedom (Xsens Technologies X-bus, Enschede, Netherlands) positioned over the T12 and S2 spinous processes. Sensors were attached to the skin using double-sided tape and the upper sensor was additionally fixed with an elastic band placed around the chest. Maximal lumbar flexion RoM was calculated as the difference in the inclination angles of the sensors in the sagittal plane. To achieve full lumbar flexion in standing position, the participants were instructed to bend forward with their knees slightly bent, imagining trying to touch their knees with their forehead while making their back as round as possible. Each participant performed two repetitions at the beginning of each test set and two at the end of each test set. The highest value of the two repetitions was used in both. The tests did not have any significant effect on RoM therefore the results of both RoM measurements at the beginning and at the end of each test set were averaged.

### Trunk stabilisation during small-amplitude trunk perturbations

**Fig 3:**
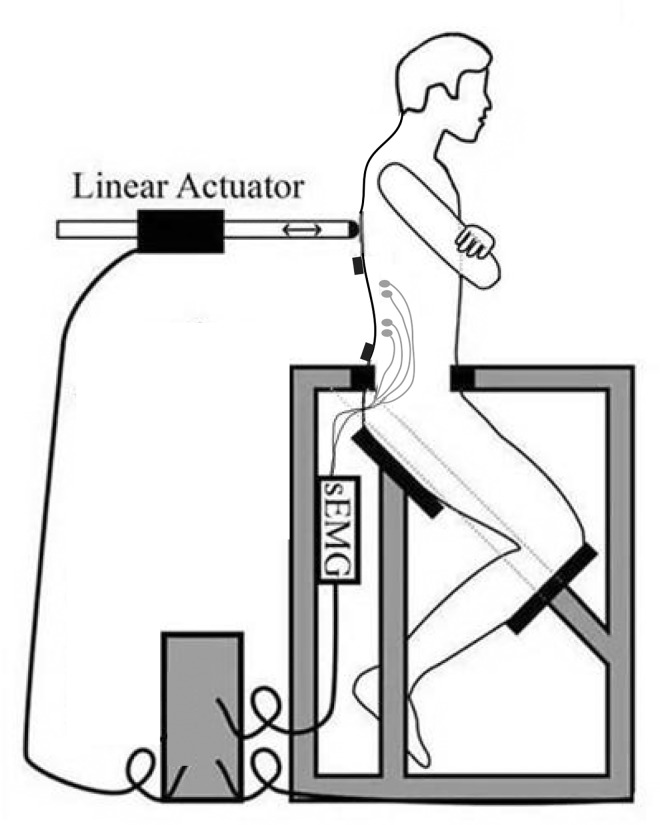
Measurements of the trunk stability in a kneeling-seated position and the perturbations were applied at the level of 10^th^ thoracic vertebrae.

Small-amplitude trunk perturbations were applied to the trunk in forward direction at the level of the T10 spinous process by means of force controlled linear actuator. The method used was proven reliable [28] and the reader is referred to previous publications for a detailed description of the procedure and related analyses [29,30]. In short, participants were positioned in a kneeling-seated position with their pelvis fixed (Fig 3). Each run consisted of a 3-s ramp force increase to 60 N of preload, to maintain contact with the participant’s back. This was followed by a 2-s static preloading, during which baseline muscle activity was determined. A dynamic disturbance (±35 N) was then superimposed on the preload. The dynamic disturbance was a crested multi sine of 20 s duration, containing 18 logarithmically spaced frequency pairs with a bandwidth ranging from 0.2 to 15 Hz, repeated twice. To reduce adaptive behaviour to the high frequency content, the power above 4 Hz was reduced to 40% [29]. Participants did not receive direct visual feedback, but if drift from the initial position was observed by the investigator (via the real time visual information on the translation of the actuator) the participant was given verbal instruction to return to the initial position. If the drift in the actuator’s position was larger than 5 cm the measurement was stopped and repeated. No feed-forward or voluntary activation was expected since the perturbations were perceived as random. In the final analysis, only the low frequencies (< 1.1 Hz) were included as the low frequency response reflects intrinsic stiffness and reflexive behaviour [29,30]. Trunk kinematics during perturbations were described in terms of translational movements of the pushing rod of the linear actuator. Closed-loop system identification was used to determine the trunk translational admittance as a frequency response function (FRF) [29–31]. The admittance describes the actuator displacement as a function of contact force. In addition, EMG time series as described above were averaged between left and right ESL muscle and related to actuator displacement to assess reflexes as FRFs. Finally, the coherence of the admittance and EMG indicates the frequency dependent input-output correlation and can attain values from 0 to 1, where 1 reflects a perfect, noise free relation. Coherence values larger than 0.18 were considered significant (p < .05) and FRFs with a coherence above 0.18 were included for further analysis [32].

### Statistical analysis

Descriptive statistics were used to report the demographic data of the participants. Log transformation, square root, square or cube transformations were used to satisfy the assumption of normal distribution, as tested with the Shapiro-Wilk test and by visual inspection of distribution plots. The assumption of sphericity was tested using Mauchly’s test and if the assumption was violated, a Greenhouse-Geisser correction was used. Analysis of variance for repeated measurements (RMANOVA) was used to check for potential differences between the control- and the pre-tests. For the RoM and body sway measurements there were two within subjects factors (*Control* (2) x *Condition* (2)) and for the perturbation parameters there were three factors (*Frequency* (5) x *Control* (2) x *Condition* (2)). Furthermore, a two-way mixed model was used to assess the reliability of the measurements. Reliability was determined within visits, comparing control tests and pre-tests separately for both visits and between visits, comparing control tests and pre-tests of both visits separately (Fig 1). RMANOVA with two within subject factors (*Condition* (2) x *Repetitions* (11)) and one between subjects factor (*Sex* (2)) was used to test for muscle activation differences during flexion between the SF and USF conditions. Similarly, RMANOVA with two within factors (*Exposure* (2) x *Condition* (2)) and one between subjects effect (*Sex* (2)) was used to assess the condition-dependent changes in RoM and in muscle activation in response to the constant force. Furthermore, to investigate the changes of admittance gain and reflex gain RMANOVA with three within subject factors (*Frequency* (5) x *Exposure* (2) x *Condition* (2)) and one between subjects factor (*Sex* (2)) was used. Significant interaction effects were followed up by *Condition* separated analysis (*Frequency* (5) x *Exposure* (2)) and further interaction effects were followed up by Bonferroni corrected pair-wise comparisons. Effects were considered significant when the corrected p < .05 and Partial Eta Squared (η_p_^2^) was used as a measure of effect size.

## Results

### Reliability

No statistically significant differences were found between measurements before and after the conditioning period. Furthermore, reliability of the RoM measurements (Table 1) was excellent within and between visits. Similarly, good to excellent within-visit and moderate to good between-visit reliability was observed for muscle activation measurements and for the parameters of neuromuscular control.

**Table 1:** Reliability results within sessions (at each visit) between control and pre-exposure test, and between test sessions for the control and pre-exposure tests.

Measurement	Visit	ICC_3,k_
RoM	Within each visit	0.97 and 0.99
	Between visits	0.93 and 0.95
EMG amplitude at 60N force	Within each visit	0.96 to 0.98
	Between visits	0.83 and 0.89
ADM gain	Within each visit	0.71 to 0.95
	Between visits	0.54 to 0.78
EMG gain	Within each visit	0.90 to 0.96
	Between visits	0.71 to 089

ICC_3,k_ = Intraclass correlation coefficient averaged measures calculated using a two way mixed model; For the admittance gain (ADM gain) and reflex gain (EMG gain) the reliability was separately calculated for each of the five input frequencies analysed.

### EMG amplitudes during intermittent flexion

There was a significant main effect of *Condition* (Table 2) on EMG amplitudes during the test, indicating higher activation during USF for both ESL (11.2 (5.4) vs 1.7 (1.4) % MVC) and ESIC (8.4 (3.4) vs 1.9 (1.4) % MVC) muscles. Although a trend towards increasing activation over repetitions in the USF condition could be seen it was not significant. There were no sex related effects.

**Table 2:** Main and interaction effects results of the RMANOVA for supported and unsupported flexion on the EMG amplitudes.

			F	df	p	η_p_^2^
Durring intermitent flexion						
mean amplitude	ESL	Condition	**134.07**	**1**	**<0.001**	**0.88**
		Exposure	0.90	10	0.491	0.05
		Condition x Exposure	1.99	10	0.113	0.10
	ESIC	Condition	**147. 89**	**1**	**<0.001**	**0.89**
		Exposure	0.51	10	0.783	0.03
		Condition x Exposure	1.25	10	0.296	0.06
Response to 60 N pushing force before and after the intermittent flexion						
mean amplitude	ESL	Condition	**13.08**	**1**	**0.002**	**0.41**
		Exposure	**53.25**	**1**	**<0.001**	**0.74**
		Condition x Exposure	**20.61**	**1**	**<0.001**	**0.52**
	ESIC	Condition	1.17	1	0.293	0.06
		Exposure	**21.15**	**1**	**<0.001**	**0.53**
		Condition x Exposure	**12.21**	**1**	**0.002**	**0.39**
median frequency	ESL	Condition	0.14	1	0.713	<0.01
		Exposure	1.46	1	0.242	0.07
		Condition x Exposure	0.01	1	0.966	<0.01
	ESIC	Condition	0.18	1	0.679	0.01
		Exposure	3.66	1	0.070	0.16
		Condition x Exposure	0.61	1	0.444	0.03

Main effects of sex and interactions with sex are not included in the Table but are described in the text when significant.

ESL – erector spinae pars lumborum; ESIC – erector spinae pars iliocostalis

**Fig 4:**
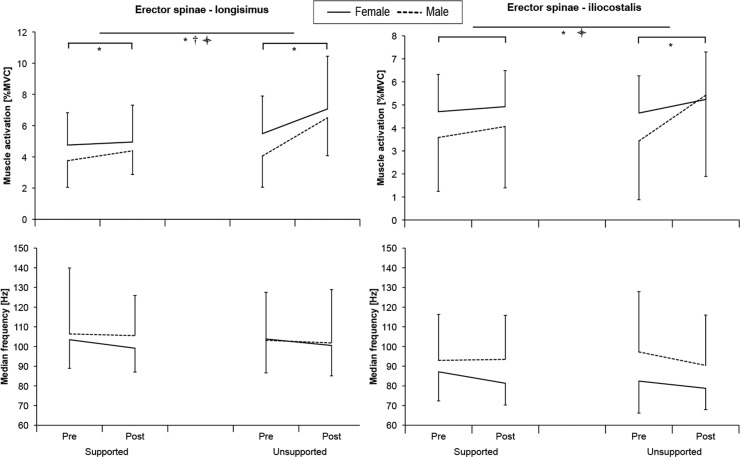
Mean muscle activation expressed as a percentage of maximal voluntary contraction and median frequency measured while resisting the 60N pushing force applied at the level of T10 spinous process before (Pre) and after (Post) intermittent flexion for male and female participants in support and unsupported conditions. *—*Time* effect (p < .05); † - *Condition* effect (p < .05); 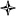
- *Interaction* effect (p < .05).

EMG amplitude during the application of the 60 N constant load showed a significant increase in both ESL and ESIC muscles (Table 2) after the intermittent flexion. The increase was significantly larger after the USF, as shown by a significant *Exposure*× *Condition* interaction effect (Fig 4). There was a significant *Exposure* × *Sex* interaction for both ESL and ESIC muscles (p < .017 F = 6.90 η_p_^2^ = .27 and p < .012 F = 7.69 η_p_^2^ = .29) and *Exposure* × *Condition* × *Sex* interaction only for the ESIC muscle (p < .012 F = 7.80 η_p_^2^ = .29), both indicating a greater increase in muscle activation in male subjects, which was more prominent after USF. There were no significant effects on the median frequency (Table 2).

### Range of motion

**Fig 5:**
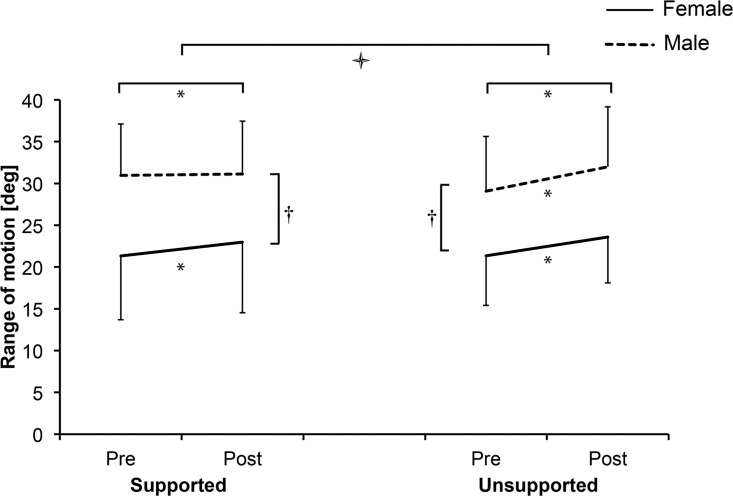
Mean range of motion (with standard deviations) before (Pre) and after (Post) intermittent flexion for male and female participants in supported and unsupported condition. *—*Time* effect (p < .05); † - *Sex* effect (p < .05); 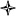
- *Interaction* effect (p < .05).

Exposure to both SF and USF resulted in a statistically significant increase in the RoM (p = .034 95% CI [0.76 – 1.75] and p < .001 95% CI [1.74 – 3.43], respectively), suggesting viscoelastic deformation of passive tissues. A significant *Exposure* × *Condition* interaction effect indicated a larger increase of RoM after USF (p = .003 F = 11.15 η^2^ = .37) (Fig 5). Female participants had significantly smaller lumbar RoM compared to male participants (p = .007, F = 9.13, 95% η_p_^2^ = .33). There also was a significant *Exposure* × *Condition* × *Sex* interaction (p = .044 F = 4.66 η_p_^2^ = .20). A pairwise comparison indicated a significant increase in RoM after SF only in female participants (p < 0.001, 95% CI [1.75 – 4.08]), while there was no change of RoM in male participants (p = 0.764, 95% CI [-0.99 – 1.33]).

### Neuromuscular control

Analysis of the neuromuscular responses to small perturbations presented good coherence ranging from .87 to .97 for admittance and from .59 to .90 for EMG-reflexes. Values exceeded the required probability level of .18, hence all measurements were included in further analyses. Testing for sex related differences did not show significant main effects of sex or interactions with sex, except for a higher increase in reflex gains in male participants regardless of condition *(Exposure* × *Sex* interaction, p = .019 F = 6.53 η_p_^2^ = .26) However, since there were no condition dependent differences between males and females the results are reported as pooled below.

**Fig 6:**
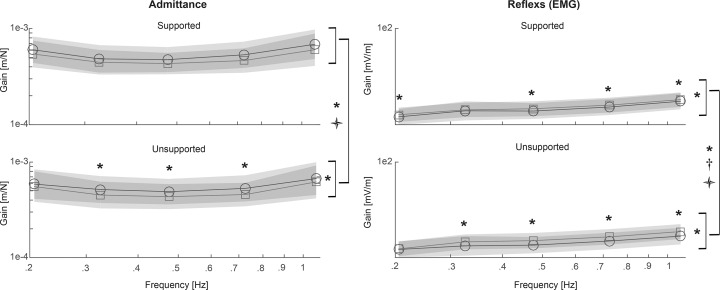
Frequency response function pre (′) and post (☐) supported and unsupported intermittent flexion averaged across all subjects. The shaded area represents the standard deviation. *—*Exposure* effect (p < .05); † - *Condition* effect (p < .05); 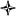 - *Interaction* effect (p < .05).

Admittance gain was reduced after intermittent flexion regardless of condition (Fig 6) as indicated by a significant main *Exposure* effect (Table 2). A significant *Condition* × *Exposure* × *Frequency* interaction was found, indicating a greater reduction of admittance gain after USF than after SF at certain frequencies (Fig 6). Further analyses separated by condition revealed a significant *Exposure* effect only in the USF condition with pairwise differences at 0.32, 0.49 and 0.75 Hz.

Intermittent trunk flexion resulted in increased reflex gains, as indicated by a significant *Exposure* main effect, and further analyses separated by condition showed an increase in reflex gain after SF and USF conditions (Table 3). Nevertheless, the significant main effect of *Condition* suggests a greater increase in reflex gains following the USF condition. Specifically, a significant *Condition* × *Exposure* × *Frequency* interaction and subsequent post-hoc testing indicated a greater increase in reflex gain after USF at all analysed frequencies but the lowest.

**Table 3:** Main and interaction effects of the RMANOVA for the gain of admittance and reflexes.

		F	df	p	η_p_^2^
**Admittance gain**					
*Condition* × *Exposure* × Frequency*		**3.10**	**2.7**	**0.039**	**0.13**
*Condition* × *Exposure*		0.00	1	0.969	0.00
Frequency*		**25.20**	**4**	**<0.001**	**0.56**
*Condition*		0.00	1	0.977	0.00
Exposure*		**9.42**	**1**	**0.006**	**0.32**
	Supported condition *Exposure*	4.12	1	0.056	0.17
	Unsupported condition *Exposure*	**5.92**	**1**	**0.024**	**0.23**
**Reflex gain**					
*Condition* × *Exposure* × Frequency*		**3.21**	**3.3**	**0.025**	**0.14**
*Condition* × *Exposure*		1.33	1	0.263	0.06
Frequency*		**179.16**	**4**	**<0.001**	**0.90**
*Condition*		**8.05**	**1**	**0.010**	**0.29**
*Exposure*		**20.59**	**1**	**0.001**	**0.51**
	Supported condition *Exposure*	**6.48**	**1**	**0.019**	**0.25**
	Unsupported condition *Exposure*	**16.26**	**1**	**0.001**	**0.45**

Main effects of sex and interactions with sex are not included in the Table but are described in the text when significant.

* Greenhouse-Geiser correction due to violation of the assumption of sphericity.

## Discussion

The aim of the present study was to assess the effects of prolonged intermittent trunk flexion on mechanical and neuromuscular functions of the trunk. In addition, we exposed the stabilising system of the spine to two different loading regimes, unsupported and supported intermittent trunk flexion, while maintaining comparable flexion angles and thus comparable loading of the ligaments. In line with the hypothesis, the lumbar RoM, muscle activation in response to 60 N force and the reflex gains increased after both SF and USF conditions with a larger increase following the latter. Surprisingly and in contrast with the hypothesis, the admittance gain decreased following prolonged intermittent trunk flexion, indicating increased resistance against trunk perturbations, which was significant only after the USF condition.

### Mechanical passive tissue deformation

Both repetitive flexion exposures used in the present study caused viscoelastic tissue deformation resulting in increased maximal lumbar flexion with significantly larger effects seen following USF. In line with previous studies [33,34], female participants presented with smaller lumbar flexion RoM. This was not expected to have an effect on the loading of the spine since both the 80% of lumbar RoM and the 35° inclination at 12^th^ thoracic vertebra level were maintained by active adjustments of the pelvic tilt. Indeed, both males and females responded similarly to the USF condition, however, after the SF condition RoM increased significantly more in female participants. The reason for this difference following the SF condition is not clear and might originate from the initially smaller RoM in female participants.

The method for RoM assessment in our study was similar to the one used in the study by Sánchez-Zuriaga and colleagues [11]. In the present study, the mean increase in RoM after SF was smaller than previously reported after one hour of continuous supported flexion at 70% of maximal RoM [11] and more similar to the increase noted after performing 100 repetitions of lifting a 10 kg load [35]. On the other hand, after USF the increase of RoM was comparable to changes reported after sustained supported flexion [11]. This is in line with earlier findings that viscoelastic deformation is larger after constant loading in comparison to cyclic loading even when the total time of loading is similar [20].

Since spinal ligaments can be assumed to be stretched to the same degree in both conditions, the results suggest that other lumbar structures are more deformed when actively maintaining near end range of trunk flexion. Dolan and Adams [35] suggested that creep and stress relaxation would occur more rapidly in the ligaments than in the disc. Sustained axial compressive loading of isolated spinal motion segment reduces the height of intervertebral disc, resulting in reduced stiffness and increased RoM of the motion segment [20]. Therefore, in our study, deformation of the intervertebral discs due to higher compressive forces might explain the larger increase in RoM after USF. On the other hand, repetitive isometric contractions of the muscle could lead to alterations of the series-elastic tissues, similar to those seen after passive stretching. Such mechanism has previously been shown in ankle flexors [36] and hip flexors [37]. It is therefore not possible to pinpoint the single structure responsible for the larger increase in RoM after USF, but the results nevertheless highlight the involvement of other structures than spinal ligaments.

### Muscle activity

Lumbar muscles were significantly more active during the flexed position periods in the USF condition. In this condition, a trend of increasing activation with repetitions can be seen. In line with that, the 60 N pushing force elicited larger post-exposure muscle activation. One of the possible explanations could be the reduction of intrinsic stiffness due to deformation of the passive viscoelastic structures [38,39]. While this mechanism could contribute to the trend of increasing muscle activity seen during the USF condition, it is on the other hand not likely to have an effect on the activation in upright position against the constant force produced by the actuator [35,40]. This is further supported by a study comparing different flexion angle exposures in which lower body tilting was applied to achieve spine flexion therefore avoiding confounding effects of different moments acting on the spine during flexion. This study showed that, with similar spinal load as in the present study, the muscle activity in the neutral position was not affected despite post-exposure differences in the intrinsic stiffness [14]. The increase in muscle activation could be a result of an increased neural drive to compensate for a reduced force production capacity of fatigued muscles [41]. The presence of muscle fatigue was supported by discomfort, which was not systematically assessed, but was frequently reported during the USF condition, but not during the SF condition.

### Neuromuscular control

The main interest of the present study was the stabilising function of the trunk muscles and passive tissues during small-amplitude perturbations. One hour of repetitive sustained flexion resulted in decreased admittance (i.e. increased resistance against perturbations) indicated by an *Exposure* main effect. Specifically, admittance decreased significantly by 5.8 to 13.9% after USF and non-significantly by 7.7 to 12.9% after SF depending on the frequency, with moderate and small effect sizes (0.23 and 0.17, respectively). Admittance at lower frequencies (< 1 Hz) is dominated by intrinsic stiffness [30] which comprises passive tissues stiffness and muscle stiffness related to background (non-reflexive) muscle activity.

In contrast with our hypothesis, the effects of fatigue were effectively compensated and decreased rather than increased admittance was found. Increased muscle activity during the 60N constant load and increased reflex gains were expected as compensations for reduced force capacity of the fatigued muscles and reduced passive tissue stiffness. However, that the net effect would reduce admittance was unexpected. These results are, however, in agreement with previous studies showing that the body successfully adapts to fatigue through an increase in background muscle activity [42]. Several authors have shown that back muscle fatigue results in increased activation of the fatigued muscles and also of antagonistic muscles indicating increased co-activation [42,43]. Increased co-activation could contribute to the decreased trunk admittance found in the present study. However, we cannot verify this as we did not measure the activation of the abdominal muscles.

In line with our hypothesis, the reflex gains increased following repetitive trunk flexion with a larger increase seen after USF. There are several possible reasons for the reflex gain increase. Firstly, the increased reflex gain could play a compensatory role in maintaining trunk stability when perturbations are applied in a state of reduced passive tissue stiffness [11,14,15]. Secondly, the larger increase following the USF condition could be related to muscle fatigue and consequently reduced force production capacity of the erector spinae muscles [44]. Finally, an increased reflex gain could arise also from increased excitability of mechanoreceptors in passive viscoelastic tissues, as has been shown immediately following prolonged spinal loading [12].

A limited number of studies addressed the consequences of trunk flexion on intrinsic and reflex contributions to trunk control. These in vivo human studies consistently reported increased reflex gains but decreased stiffness after creep deformation [22] and stress relaxation [14] of passive viscoelastic spinal tissue induced by trunk flexion. In contrast, in the present study, the stiffness increased in both flexion conditions. However, it should be noted that in our study the admittance gain reflected combined intrinsic stiffness and reflex contributions. The other main difference between the present and previous studies was the duration of exposure to the flexed position, which was much longer in the present study. Possibly changes in trunk stiffness related with trunk flexion are time-varying. This possibility was previously suggested by Parkinson and colleagues [45] showing a trend of reduced passive stiffness following 30 minutes of cyclic trunk flexion, which was reversed following the consecutive 30 minutes. In their study, only passive tissue contributions were considered and the muscle activation was monitored only to exclude the trials when muscle activation increased more than 5% during the assessment. However, according to present study, muscle activity variations within this range should also be considered as these could substantially contribute to the trends noted by the researchers [46].

Increased muscle activity of the posterior muscles has repeatedly been shown during the recovery phase following creep loading of the spinal ligaments of anesthetised cats. This hyper-excitability has been attributed to the presence of acute inflammation due to the micro-damage of the ligaments [13,21]. The model of pain development introduced by Solomonow [13] differentiates between low loading, where post-exposure excitability did not exceed the pre-exposure values, and high loading, where post-exposure excitability exceeded initially measured muscle activation typically after 2 to 3 hours. Although the paraspinal muscle excitability was reduced during cyclic loading of spinal ligaments, some initial increase in excitability, immediately following 20 minutes of loading, which did not exceed the initially measured muscle responses, was also shown [13]. In contrast, another study showed that the initial hyper-excitability can exceed pre-exposure values following 60 minutes of creep loading of spinal ligaments [12]. Furthermore, cumulative effects of spinal loading could be prevented with a recovery period of equal duration as the duration of the loading if the loading period was 30 minutes or less. It was concluded that longer loading of the spine exceeding a certain threshold can cause micro-damage and is sufficient to trigger an acute inflammation [12]. This work further supports the time-varying and intensity dependent nature of effects of prolonged loading of the spine.

Hyper-excitability of trunk muscles due to the acute inflammation would explain both the increased baseline activity resulting in increased spinal stiffness as well as increased reflex gains seen in the present study following trunk flexing in both conditions. Although ligaments were loaded similarly in both conditions, a significant decrease of the admittance was noted only after USF. Taking this into account and also the fact that the perturbations were applied in upright neutral position therefore inducing small lumbar movements within the neutral zone, yielding low stress in spinal ligaments, one can assume that hyper-excitability in this case originated from other structures than spinal ligaments.

### Limitations and conclusions

There are a few limitations of the present study. Firstly, a convenience sample was recruited by means of personal communication and social media. As a consequence, relatively young participants were included, which limits the generalizability of the results. Secondly, the measurements were performed in the morning and afternoon, therefore some circadian influence could be expected [47]. To minimize these effects, the participants were scheduled for the measurements at a similar time of the day for both conditions (visits). Furthermore, participants had to maintain the requested active position in the USF condition with the help of real-time visual feedback. The requested position was “unnatural” for some participants and therefore they probably activated trunk muscles somewhat more than they would in their preferred flexed position. Two male participants (one in each condition) did not have a control set of tests at the first visit, but this did not affect the final results. Lastly only the immediate effects were investigated therefore in future studies, the recovery after longer exposure to trunk flexion would be of interest. Furthermore, studies in real working environments that require prolonged sustained and/or repeated flexion are needed to elucidate the effects of repeated exposure to realistic occupational exposure to spinal loading.

To conclude, the present study has shown that one-hour of intermittent trunk flexion increases trunk range of motion, but decreases trunk admittance and increases reflex gains. The change in admittance is in contrast with results of previous studies that used shorter lasting interventions, therefore supporting the idea of a time-varying response to lumbar viscoelastic deformation. Therefore, the duration of the spinal loading should be considered when assessing cumulative low back loading and its effects. The effects of trunk flexion were similar but significantly smaller when external passive support for the upper body was used. For this reason, the use of upper body support can be recommended in occupational settings requiring flexed postures.
